# Intracerebral Hemorrhage and Reversible Cerebral Vasoconstriction Syndrome in a Patient With COVID-19

**DOI:** 10.7759/cureus.17408

**Published:** 2021-08-24

**Authors:** Aswin Srinivasan, Branden C Wilson, Matthew Bear, Ammar Hasan, Obadah Ezzeldin, Salman Alim, Samer Elfallal, Xiang Fang, Mohamad Ezzeldin

**Affiliations:** 1 Internal Medicine, HCA Houston Kingwood/University of Houston College of Medicine, Kingwood, USA; 2 Diagnostic Radiology, University of Texas Medical Branch, Galveston, USA; 3 Critical Care Medicine, HCA Houston Kingwood/University of Houston College of Medicine, Kingwood, USA; 4 Neurosurgery, HCA Houston Kingwood/University of Houston College of Medicine, Kingwood, USA; 5 Neurology, University of Texas Medical Branch, Galveston, USA; 6 Neuroendovascular Surgery, HCA Houston Kingwood/University of Houston College of Medicine, Kingwood, USA

**Keywords:** reversible cerebral vasoconstriction syndrome, intracerebral hemorrhage, covid-19, sars-cov-2 (severe acute respiratory syndrome coronavirus -2), convexity subarachnoid hemorrhage

## Abstract

Neurological manifestations, such as encephalopathy, intracranial neuropathy, headache, and cognitive decline, are often presented in patients with COVID-19 infection. Since the onset of the pandemic, acute ischemic stroke associated with a hypercoagulable state caused by COVID-19 is increasingly being reported. Hemorrhagic stroke is also reported via poorly understood mechanisms. We report one of the first-ever cases of intraparenchymal hemorrhage, subarachnoid hemorrhage secondary to reversible cerebral vasoconstriction syndrome in a patient with COVID-19 infection.

## Introduction

Reversible cerebral vasoconstriction syndrome (RCVS) is a condition characterized by reversible multifocal narrowing of cerebral arteries [1]. Patients commonly present with sudden onset severe headache. The condition may result in focal neurologic deficits secondary to ischemic or hemorrhagic stroke or subarachnoid hemorrhage (SAH) [1,2]. The pathophysiology of this condition is not fully understood but is associated with vasoconstricting drugs and the postpartum period. The provoking factors for RCVS are identified in approximately 25%-60% of cases [2]. However, RCVS can occur spontaneously as well. The postpartum state is the most important contributor accounting for 50%-60% of cases. Some other identified triggers include vasoactive drugs such as sympathomimetic drugs, migraine abortive medications, interferon treatment, NSAIDs, alcohol, blood transfusions, erythropoietin, intravenous immunoglobulin, migraine and trauma [3].

COVID-19 is a pandemic disease caused by infection with SARS-CoV-2. One of the factors leading to the pandemic spread of this virus is that many people transmit the virus before symptom onset or never develop symptoms. The most common symptoms are acute hypoxic respiratory failure, progressing to acute respiratory distress syndrome (ARDS) in a significant minority of patients. Neurologic symptoms are varied; many are related to hypercoagulability associated with the viral infection [4]. In this case report, we present a rare case of intracerebral hemorrhage (ICH) and SAH secondary to RCVS in a patient with SARS-CoV-2 infection.

## Case presentation

**Figure 1:**
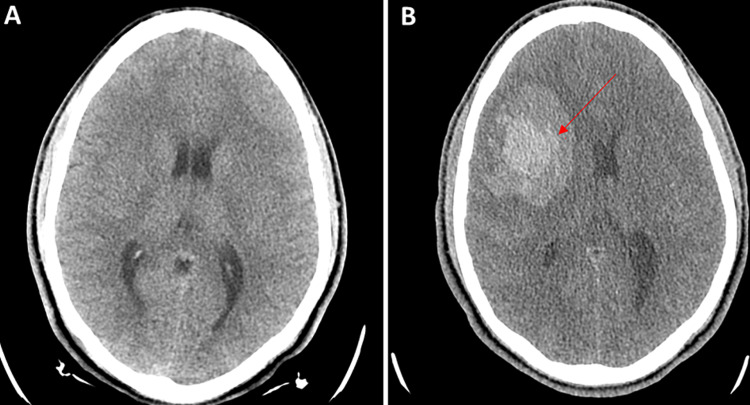
(A) Axial cut, non-contrast head CT on admission was unremarkable. (B) Axial cut, non-contrast head CT four hours after admission demonstrated 6.4 x 4.4 x 4 cm right fronto-parietal intracranial hemorrhage with mass effect and 7 mm right to left midline shift. Subarachnoid hemorrhage in the right frontal and temporal lobes.

**Figure 2:**
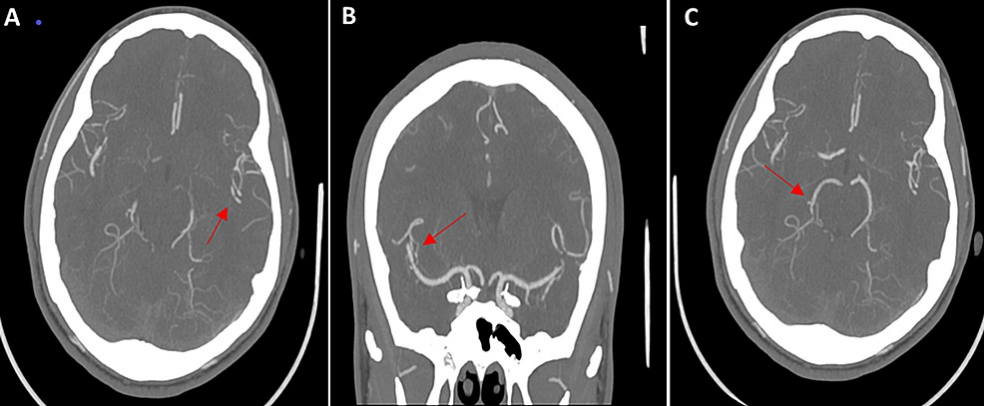
(A) Axial cut, CT angiogram of the head demonstrating focal areas of stenosis involving the left inferior M2 middle cerebral artery (MCA). (B) Coronal cut, CT angiogram of the head demonstrating focal areas of stenosis involving the right superior M2 MCA. (C) Axial cut, CT angiogram of the head demonstrating focal areas of stenosis involving the right P2 posterior cerebral artery.

**Figure 3:**
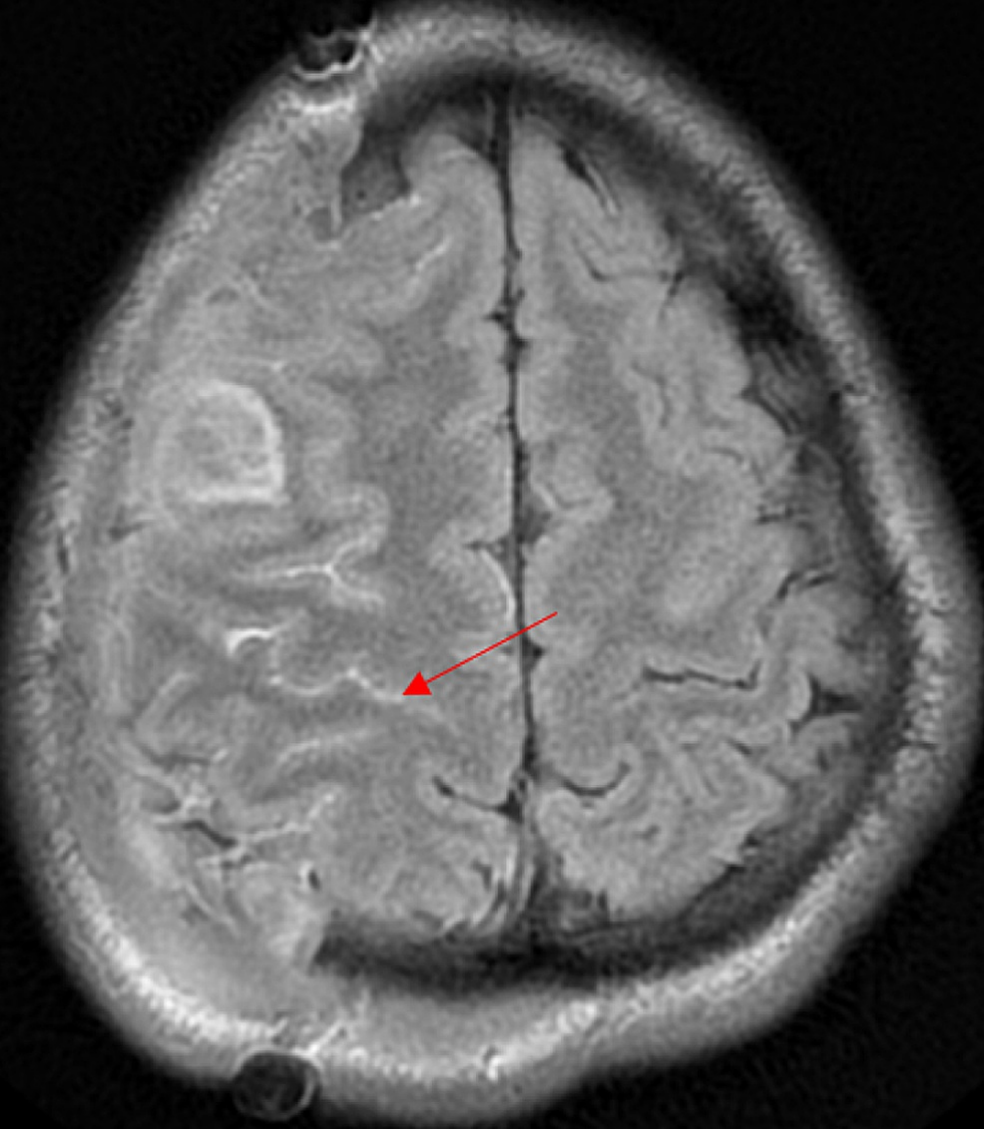
MRI brain with and without contrast post decompression, fluid-attenuated inversion recovery (FLAIR) sequence demonstrating subarachnoid hemorrhage in the right frontal and parietal lobe.

An 18-year-old African American male with a past medical history of asthma presented to the emergency department with a thunderclap headache, nausea and vomiting for the past two days. The blood pressure at admission was 149/79 mmHg, and there were no focal deficits on neurological examination. Blood tests were grossly unremarkable including platelet count, coagulation panel and C-reactive protein (CRP). Initial CT head without contrast showed no acute intracranial abnormality (Figure 1A). CT angiogram of the head showed multifocal areas of stenosis, predominantly involving M2/M3 segments of a bilateral middle cerebral artery (MCA) and P2/P3 segments of the bilateral posterior cerebral artery (PCA), A2 segment of the right anterior cerebral artery (ACA), without large vessel occlusion (Figures 2A-2C). Four hours after the initial presentation, the patient became unresponsive with a Glasgow coma score (GCS) of 3. STAT repeat CT head without contrast revealed a 6.4 x 4.4 x 4 cm (66 cc) hemorrhage in the right frontal lobe with 7 mm of leftward midline shift, mass effect on the right lateral ventricle, and SAH in the right frontal and temporal lobes (Figure 1B). The ICH score was 3 with 72% mortality. After transferring to ICU, the patient was intubated. Subsequently, emergent right decompressive craniectomy was performed and the patient was transferred to the neurocritical care unit. Subsequent MRI brain with and without contrast (Figure 3) ruled out any clear etiology that would explain the patient intracranial and SAH.

**Figure 4:**
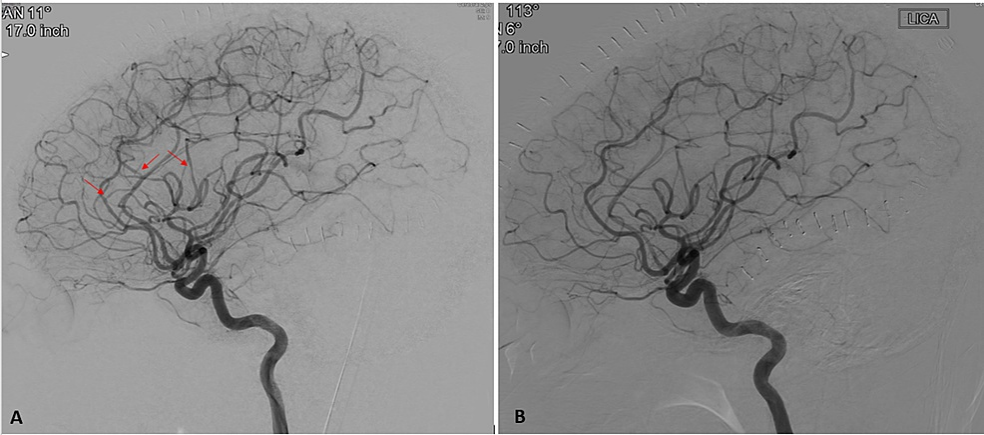
(A) Left internal carotid artery angiogram (lateral view) demonstrating mild to moderate diffuse focal stenoses involving the left MCA and bilateral ACAs. (B) Left internal carotid artery angiogram (lateral view) demonstrating near-complete resolution of previously seen diffuse focal stenoses.

Vasculitis evaluation including antinuclear antibody (ANA), C-reactive protein (CRP), sedimentation (SED) rate, anti-neutrophil cytoplasmic antibody (ANCA) and serum neurosyphilis antibody was negative. Urinary drug screen (UDS) revealed the presence of 9-THC. Conventional cerebral angiogram revealed mild to moderate diffuse focal intracranial stenosis concerning RCVS (Figure 4A). 80-mg of IV verapamil every 8 hours was initiated to treat the RCVS. The patient was successfully extubated on day 11 of hospitalization. Repeat cerebral angiogram on hospital day 13 showed almost near-complete resolution of the previously seen diffuse focal stenosis (Figure 4B) supporting the diagnosis of RCVS. Verapamil was slowly weaned off the following days. The patient was later transferred to an inpatient physical rehabilitation facility for two weeks of treatment and eventually made a full functional recovery over the course of the following three months.

## Discussion

RCVS is a medical condition in which there is multifocal arterial constriction and dilation in the cerebral vasculature [1]. This diagnosis should always be considered in the setting of recurrent thunderclap headache as it is the most common clinical manifestation. The majority of RCVS patients have normal initial CT scan of the brain, but some present with SAH and/or ICH [5]. RCVS most commonly affects women. Risk factors include recreational drugs, alcohol, vasoactive medications, hypercalcemia postpartum period, and sexual activity [3]. The pathogenesis of RCVS remains uncertain but autonomic dysregulation, oxidative stress and genetic predisposition have been postulated [2].

Complications of RCVS include ICH, SAH, posterior reversible encephalopathy syndrome (PRES) and ischemic infarction. Brain hemorrhage in RCVS tends to occur within the first three days. One cohort found hemorrhage (ICH and SAH) in 43% of RCVS patients with SAH being more common than ICH [6]. Infarction tends to occur with cases of severe proximal vasoconstriction. No particular trigger or risk factor was associated with hemorrhagic compared to non-hemorrhagic cases of RCVS [6]. In one prospective series of 67 patients, the incidence of cortical SAH (cSAH) was 22% and ICH 6% [7]. These were reported to be an early complication of RCVS, occurring within the first week [7]. In another cohort, Muehlschlegel et al. reported that patients with RCVS-associated SAH (RCVS-SAH) have a higher incidence of CT hypodensities at admission than patients with aneurysmal associated SAH (aSAH) or cSAH [7]. RCVS-SAH was typically located in the hemispheric convexities as opposed to blood in the Sylvian fissure or basal cisterns commonly observed in aSAH and cSAH [8]. RCVS-SAH had less severe neurological presentations and lower Hunt-Hess grades at presentation than did patients with aSAH [8].

RCVS is generally not associated with a viral infection. One case of RCVS in a patient with COVID-19 infection has been reported [9]. Dakay et al. report a young female patient with COVID-19 infection who presented with a thunderclap headache who was found to have bilateral high frontal SAH on non-contrast CT brain. Interestingly, diagnostic cerebral angiogram demonstrated significant focal vasospasms in the anterior circulation suggesting RCVS [9]. Another reported case of ICH in a COVID-19 patient was that of a 75-year-old woman who was hospitalized for respiratory failure and on triple therapy (aspirin, clopidogrel and enoxaparin). This patient died as a result of large SAH and IPH [10]. The case we present is unique in that the patient is young and not on medications that increase bleeding risk; moreover, the patient made a full functional recovery.

The etiology of RCVS in COVID-19 is under investigation. SARS-CoV-2 infection is mediated by angiotensin-converting enzyme 2 (ACE2) receptors, which are expressed in several organs, including the lung, kidney, and small intestine [11,12]. ACE2 receptors are also highly expressed on vascular endothelium and arterial smooth muscle cells [12]. The vascular endothelium plays a crucial role in the regulation and maintenance of vascular tone in all organs [11,12]. In one post-mortem analysis, Varga et al. reported the presence of viral elements within endothelial cells with evidence of endothelial and inflammatory cell death in several organs, suggesting an important role of endothelial cell injury in patients with COVID-19 [11, 12]. Endothelial cell injury secondary to SARS-CoV-2 infection is likely a key player in microvascular dysfunction by interrupting the regulation and maintenance of vascular tone [11]. Direct local effect of SARS-CoV-2 on ACE2 receptors leading to endothelial cell dysfunction and vasoconstriction with the concomitant local inflammatory release of vasoactive substances causing vasodilation and loss of cerebral autoregulation in the brain could result in RCVS. SARS-CoV-2-induced vasoconstriction with concomitant downregulation of ACE2 receptors resulting in vasodilation and dysregulation of the renin-angiotensin axis leading to loss of cerebral autoregulation is another postulated mechanism [9].

To our knowledge, we report the first case of large ICH and SAH secondary to RCVS in the setting of COVID-19 infection. Our case is unique in that initial CTA and traditional cerebral angiography showed diffuse cerebral vasoconstriction, which resolved on subsequent angiography, and after treatment with verapamil. This case adds to the growing evidence of a possible association between RCVS and SARS-CoV-2 infection. RCVS is generally associated with SAH but this case presentation is atypical in nature due to the association of RCVS with large ICH. In addition, our patient did not have increased systemic inflammatory response or prothrombotic serum markers. SARS-CoV-2-related microangiopathic damage secondary to either direct viral damage or a local immune response leading to dynamic vessel wall changes could explain cerebrovascular events such as RCVS. Our patient suffered non-aneurysmal SAH and a rare complication of parenchymal hemorrhage in the frontal lobe. This patient’s RCVS2 score is 9 (5 points for single thunderclap headache, 3 points for vasoconstrictive trigger identified, 1 point for SAH present on imaging), with a score of >5 having high specificity and sensitivity (99% and 90% respectively) for diagnosing RCVS [13]. One limitation of this case study is that urine toxicology screens were positive for cannabinoids, which could be an exacerbating factor for RCVS in our patient. A recent systematic review by Short et al. reported strong evidence for cannabis as a precipitating factor for RCVS than other illicit drugs [14].

## Conclusions

There is a need to consider ICH secondary to RCVS as a potential neurological sequelae of SARS-CoV-2 infection. We present one of the first case reports of a large ICH and SAH secondary to RCVS in a patient with COVID-19 infection. As our understanding of this infection and its clinical manifestations continue to grow, it is important to recognize the unusual and atypical extra-pulmonary manifestations. 
